# Improving Outcomes After Relapse in Ewing's Sarcoma: Analysis of 114 Patients From a Single Institution

**DOI:** 10.1155/SRCM/2006/83548

**Published:** 2006-11-06

**Authors:** Anne M. McTiernan, Anna M. Cassoni, Deirdre Driver, Maria P. Michelagnoli, Anne M. Kilby, Jeremy S. Whelan

**Affiliations:** Department of Oncology, University College Hospital, 250 Euston Road, London NW1 2PG, UK

## Abstract

The outcome for patients with relapsed Ewing's sarcoma is poor. A retrospective analysis was carried out to identify factors associated with improved survival. Between 1992 and 2002, 114 patients presented with relapsed or progressive disease. Median time to progression/relapse was 13 months (range, 2–128). Treatment at relapse included high dose treatment (HDT) in 29 patients, and surgery or definitive radiotherapy in 29. 2 and 5-year post relapse survival (PRS) was 23.5% and 15.2%, respectively. In multivariate analysis, the most significant factors associated with improved survival were disease confined locally or to the lungs (2-year PRS, 40% versus 6%; *P* < .001), relapse > 18 months from diagnosis (2-year PRS, 53% versus 8%; *P* < .001), HDT at relapse (2-year PRS, 62% versus 11%; *P* < .001), and surgery and/or radiotherapy at relapse (2-year PRS, 51% versus 14%; *P* < .001). First treatment failure in Ewing's sarcoma is mostly fatal. Improved survival can be achieved in selective patients with aggressive treatment. These improvements are confined to those without bone or bone marrow metastases.

## INTRODUCTION

Though still biologically enigmatic, Ewing's sarcoma family of tumours (ESFT) has now been repeatedly demonstrated to be curable cancers. Results from large studies of first-line combined modality treatments indicate that perhaps two thirds of patients will be long-term survivors. However, for patients with relapsed or progressive disease, the prognosis is very poor, with only a small proportion of patients being cured [1–3]. In order to identify the factors that might be associated with improved survival in patients with relapsed or progressive ESFT, we carried out a retrospective analysis at our institution, together with a review of the literature.

## PATIENTS AND METHODS

### Patients characteristics

Between 1992 and 2002, 61 males and 53 females, median age 19 (range, 4–48), presented to the London Bone and Soft Tissue Sarcoma Service with relapsed or progressive ESFT. The patient characteristics are given in Table 1. Fifty-three patients had metastases at diagnosis, of whom 29 had metastases confined to the lungs. Primary site was of the extremities in 56 patients, pelvis in 30 patients, and central (nonpelvic) in 26. Two other patients had multifocal tumours. There were 87 primaries in bone and 27 soft-tissue tumours. The median tumour volume, assessed in 86 patients, was 255 mL (range, 50–3500 mL). Diagnosis had been established by morphology and immunocytochemistry in all patients, with no routinic use of molecular diagnostic techniques. Confirmation of recurrence by biopsy was undertaken whenever possible.

### First-line treatment

Details of first-line chemotherapy are given in Table 1. The majority of patients (90%) had been treated in accordance with successive European protocols for ESFT, with those diagnosed before 1993 being treated according to the ET-2 protocol [4]; those diagnosed between 1993 and 1997 being treated according to the Eicess' 92 protocol [5]; and patients diagnosed from 1998 onwards being treated with 6 cycles of vincristine, ifosfamide, doxorubicin, and etoposide, followed by consolidation treatment with either 8 cycles of vincristine, actinomycin, and ifosfamide or cyclophosphamide, or high-dose therapy (HDT) with busulphan and melphalan [6], in accordance with the current EURO-EWING 99 protocol (ISRCTN:61438620). All but 3 patients received doxorubicin and all but 4 patients received ifosfamide as part of first-line treatment. Fourteen patients received HDT as consolidation of first-line therapy.

#### Local treatment

Local treatment was surgery alone in 34 patients, surgery and adjuvant radiotherapy in 20 patients (2 patients with preoperative radiotherapy), and radiotherapy alone in 46 patients. Fourteen patients had no local treatment due to widespread bone metastases in 3 patients (all 3 received HDT as part of first-line treatment), disease progression in 8 patients, persistent bone marrow disease in 2, and patient refusal in the presence of widespread metastatic disease in 1. One further patient underwent pulmonary metastatectomy for persistent pulmonary metastases at the end of first-line treatment.

### Statistical methods

Survival after first treatment failure, termed postrelapse survival (PRS), was calculated from the date of PD or recurrence to the date of death or date of last followup. The duration of first event-free survival (EFS1) was calculated from the date of diagnosis to the date of first treatment failure. PRS was estimated using Kaplan-Meier methods [7]. The significance of influencing factors was tested by the log-rank test. In the case of more than two curves, the overall *P* value was given. Factors considered included demographic factors (age at diagnosis, gender, and age at relapse), diagnostic factors (primary site, metastases at diagnosis, tumour volume), local treatment in first-line therapy, factors at relapse (timing of relapse and site of relapse), and treatment at relapse. Multivariate analysis was carried out using Cox's proportional hazards model [8], with all variables found to be significant in univariate analysis being entered into a multivariate model using a stepwise procedure.

## RESULTS

### Details at relapse/progressive disease

Thirty-seven patients developed PD within first-line treatment and 77 recurrent disease. The median time to first treatment failure was 13 months (range, 2–128 months) with 42% of events occurring within the first 12 months. Site of first relapse or progression is given in Table 1.

### Treatment of relapse

#### Chemotherapy

Eighty-nine patients (78%) were treated with chemotherapy at relapse, 12 with palliative intent only. Chemotherapy details at relapse are given in Table 2. Several different regimens were employed reflecting patient factors (major organ function, duration of remission, need for mobilisation, and collection of stem cells) and evolving practice. Patients were often treated with more than one regimen in an attempt to reduce disease burden to the minimum achievable one. The majority of patients were treated with second-line regimens, principally carboplatin-based chemotherapy [9]. When short durations of response with carboplatin regimens were observed, and in response to others' data, high-dose ifosfamide, at a median dose of 15 g/m^2^ (range 12 g/m^2^–18 g/m^2^), was employed [10]. Twenty-two patients, the majority of whom relapsed more than 6 months from the end of first-line treatment, were initially retreated with an ifosfamide/doxorubicin-based regimen, to maximise exposure to anthracycline-based chemotherapy. Twenty-five patients were not treated with chemotherapy at relapse or progression, due to either poor tolerance in the presence of widespread refractory disease or rapid relapse after HDT, or patient choice.

#### High dose therapy at relapse

Twenty-nine of 77 patients (38%) treated with curative intent at progression/relapse were treated with high-dose chemotherapy after second-line chemotherapy. Patients were treated with second-line treatment until remission was achieved or there was no further response. HDT comprised of busulphan and melphalan in 19 patients, and other regimens in 10. For those who proceeded to HDT at relapse, the median time from diagnosis to relapse was 23 months (range, 3–70 months). The best response at time of HDT was CR in 13 patients, PR in 8, responding disease in 7, and progressive disease in 1 patient. This latter patient had operable lung metastases at relapse and was therefore deemed suitable for HDT, despite disease progression. All other patients treated with curative intent were initially considered for HDT, but did not receive it principally due to inadequate response to second-line chemotherapy, rapid progression of initially responding disease, insufficient major organ function, or patient preference. The probability of a patient receiving HDT at relapse remained constant over the 10-year period (*P* = .589).

#### Surgery

Twenty-one patients (18%) had surgery as part of second-line treatment, although for one patient this was for palliative amputation of local recurrence only. Fifteen underwent excision of a local recurrence (4 with adjuvant radiotherapy), achieving second complete remission in 11 patients, and 4 with disease elsewhere, in anticipation of proceeding to HDT. Metastatectomies were performed in 5 patients, all with complete macroscopic excision. Three patients underwent lung metastatectomy (one with adjuvant whole lung irradiation), 1 patient had a cerebral metastatectomy, and 1 patient underwent excision of a para-spinal mass. In total, complete macroscopic resections were achieved in 16 of the 20 patients.

#### Radiotherapy

Sole definitive radiotherapy for relapse was given to 8 patients. In 4 patients this was for local recurrence deemed unresectable (3 extremity tumours, 1 chest wall). Three patients, 1 of whom also underwent HDT, received definitive radiotherapy to isolated bone metastases, and 1 patient with 2 sites of distant metastases received radiotherapy to both sites following TBI (given as part of HDT) to achieve a radical dosage to both sites of disease.

### Survival

#### Postrelapse survival

At 1st April 2005, the median followup for the whole group was 67 months (range, 0–129). At this time 14 patients (12.3%) remained alive continuously disease free, with a median followup of 61 months from first relapse (range, 35–129 months). Another patient was alive and disease free following resection of a further loco-regional recurrence 58 months after first relapse, and a second patient had become lost to followup with uncontrolled disease 19 months from relapse.

Three patients (2.6%) died of causes related to further treatment. One died of pneumocystis carinii during chemotherapy for a second relapse having previously been treated with HDT; one patient died of gastrointestinal toxicity 3 months after HDT; and one patient suffered radiation pneumonitis with lung abscesses and chronic infection after HDT and lung irradiation, dying 18 months later. Recurrent disease was suspected but not proven to be present.

**Figure 1:**
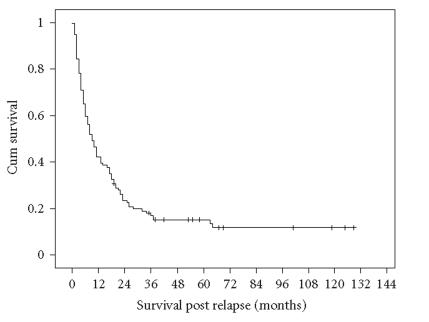
Postrelapse survival according to all patients (*n* = 114).

The median estimated PRS for the whole group was 9 months (range 0–129), with the 2- and 5-year estimated PRS being 23.5% (±4.0%, standard error) and 15.2% (±3.4%), respectively (Figure 1).

### Factors influencing survival

Table 3 lists the PRS according to age, gender, primary site, metastases at diagnosis, relapse details, and treatment at relapse.

#### Factors at diagnosis

No influence on PRS was evident for gender, age at diagnosis, age at relapse, primary site, or tumour volume (Table 3). When grouped together, patients with metastases at diagnosis had a poorer PRS compared to those with no metastases, but this was largely accounted for by the poor survival of 24 patients with extra-pulmonary metastases at diagnosis (Table 3).

#### Factors at relapse

Patients with local recurrence or lung metastases at relapse (with or without local recurrence) had superior survival compared to those with extra-pulmonary recurrence. Moreover, patients with disease confined locally or to the lungs at both diagnosis and relapse had superior survival to those with extra-pulmonary disease at any time. Relapse within the first 18 months was also associated with inferior survival (Table 3).

#### Treatment at relapse

**Figure 2:**
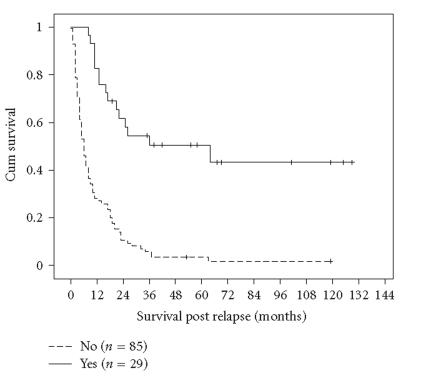
Postrelapse survival according to high-dose therapy (HDT) at relapse (*P* < .001).

Whether patients received HDT or not at relapse was the strongest predictor of survival in univariate analysis, with a 5-year PRS of 50.6% (±9.5%) for those who received HDT compared to 3.5% (±2.0%) for those who did not (*P* < .001; Figure 2). The ability to perform definitive local treatment to the site of relapse was also a strong predictor of outcome, with no difference seen between those who received surgery or those who received radiotherapy (Table 3).

#### Multivariate analysis

When considered in multivariate analysis, the strongest factors associated with superior survival were disease confined either to the primary site or to the lungs at both diagnosis and relapse; relapse greater than 18 months from diagnosis; HDT at relapse; and definitive treatment to the site of relapse (Table 4).

## DISCUSSION

This report describes the outcome of a large series of patients with ESFT after failure of first-line treatment. Although this is a retrospective analysis, the data presented is especially valuable as it reflects relatively recent practice in this disease, as this large number of patients has all been treated within a 10-year period. Although the outcome for the group overall is poor, there are a notable number of long-term survivors, and important and consistent factors associated with improved survival have been identified.

The 5-year PRS survival of 15.2% in this group of patients is in accordance with that described elsewhere. Reports detailing studies of first-line treatment describe a PRS between 0 and 22% [11–18], although many of these studies include only patients with localised disease at diagnosis (Table 5). Time to first relapse has commonly been found to be a significant factor in influencing PRS, with those who relapse within 2 years of diagnosis having an inferior survival compared to those who relapse beyond 2 years [1–3, 15, 17, 19, 20]. In this study we found that a cutoff of 18 months was a stronger predictor of survival, those relapsing after 18 months having an estimated 5-year PRS of 32%. When disease progression occurs during first-line treatment subsequent survival is rarely reported. However 3 of 37 patients described in this study are long-term survivors, indicating that long-term remissions can occasionally be obtained with intensive treatments in selected patients with early progression.

Only a small number of studies have specifically examined survival after relapse in ESFT (Table 6). From these studies, factors associated with superior survival included relapse (rather than progression within first-line treatment) [21]; local recurrence only [1]; lung metastases rather than extra-pulmonary disease [1, 3]; definitive surgery and/or radiotherapy to the site of relapse [1, 3]; response to second-line therapy [22]; HDT at relapse [22]; and relapse more than 2 years from diagnosis [1–3, 20, 22]. Where stated, 5-year PRS from these studies ranged from 13.8% to 20.0%, (Table 6).

The survival of the cohort reported here compares favourably with these series, as the analysis was not confined to those with just localised disease at diagnosis and all had been treated with modern first-line regimens. A key goal in analysing and reporting this series was to identify treatment factors that might improve survival after relapse of ESFT. The results indicate the importance of an aggressive approach to local failure and, in particular, suggest a role for HDT in improving PRS, previous evidence for which has principally been derived from registry reports [23, 24].

In this study, benefit of intensive treatment was confined to those with either local disease or lung metastases. It is therefore possible that a benefit similar to that reported for HDT might have been achieved had lung irradiation been used instead. This however seems unlikely as other evidence indicates only a small effect from the use of lung irradiation [25] and also when second-line treatment for ESFT fails, disease rarely remains confined to the lungs.

A limitation of all these studies is that as retrospective analyses it is not possible to control for selection bias. In both this series and that conducted by Barker et al [22], it is probable that some of the long-term survivors may have achieved the same status without the use of HDT. However it is unlikely that this would be the case for all the survivors in the series reported here. In the absence of evidence from prospective randomised studies of HDT in patients with relapsed ESFT, which would present insuperable challenges to conduct, consolidation of response with HDT would appear to be an appropriate goal where possible, given the otherwise uniformly dismal outlook for patients with relapsed disease.

## Figures and Tables

**Table 1:** Patients characteristics. (Abbreviations: V = vincristine; D = doxorubicin; I = ifosfamide, E = etoposide, A = actinomycin-D, C = cyclophosphamide; HDT = high-dose therapy; PD= progressive disease.)

	*No (%)*

*Median age at diagnosis:*	19 years (range 4–48)

Male	61 (53.5)
Female	53 (46.5)

*Site of primary:*	

Extremity	56 (49.2)
Axial/Central	
Pelvis	30 (26.3)
Trunk	22 (19.4)
Other^(a)^	4 (3.5)
Multifocal	2 (1.8)

*Size of primary tumour:*	

< 100 mL	73 (64.0)
> 100 mL	13 (11.4)
Unknown	28 (24.6)

*Metastases at diagnosis:*	

None	61 (53.5)
Lung only	29 (25.4)
Bone ± other, (excluding bone marrow)	8 (7.0)
Bone marrow ± other	14 (12.3)
Lymph node	2 (1.8)

*First-line chemotherapy protocol:*	

ET-2 (VAID)	17 (14.9)
Eicess' 92 (VAID ±E ± C)	50 (43.9)
EE99 (EVAID ±C ± HDT)	36 (31.2)
P6 (VDCIE)	5 (4.4)
MMT'89 (VAI or VAC)	3 (2.6)
VDC	2 (1.8)
ID	1 (< 1)

*Site of first relapse/PD:*	

*Local*	29 (25.4)
*Distant*	
Lung only	32 (28.1)
Extrapulmonary	33 (28.9)
Combined	
Local + lung only	8 (7.0)
Local + extrapulmonary	12 (10.5)

^(a)^ Other: scalp = 1, sphenoid = 1, mandible = 1, neck = 1, multifocal = 2.

**Table 2:** Definitive treatment at relapse. (Abbreviations: RT = radiotherapy; HDT = high-dose therapy; TBI = total body irradiation.)

Treatment	No

*Chemotherapy (n* = 89)	

– Carboplatin, etoposide, cyclophosphamide	26
– Ifosfamide, doxorubicin, +/− actinomycin,	22
+/− vincristine, +/− etoposide	
– High-dose ifosfamide (12–18 g/m^2^)	18
– Other^(a)^	11
– Palliative only	12

*Surgery (n* = 20)	

– Excision of local recurrence (no RT)	11
– Excision of local recurrence + RT	4
– Metastatectomy: Lung	3
Cerebral	1
Para-spinal mass	1

*Definitive radiotherapy (n* = 8)	

– Local recurrence	4
– Bone metastases	4

*HDT (n* = 29)	

– Busulphan + melphalan	19
– Melphalan + etoposide (+ TBI, 3 patients)	7 (3)
– Melphalan (+ TBI, 1 patient)	2 (1)
– Busulphan + cyclophosphamide	1

^(a)^ Other: ifosfamide + etoposide = 3; cisplatin + etoposide ± vincristine = 3; vincristine + doxorubicin + cyclophosphamide = 3; cyclophosphamide + topotecan = 1; ifosfamide + vincristine = 1.

**Table 3:** Summary of univariate analysis. (Abbreviations: SE = standard error; EFS1 = first event free survival; NR = not reached; RT = radiotherapy.)

Variable	Grouping	No of patients	% Postrelapse survival (±1 SE)		*P* value

			2-year	5-year	(log rank)

*All patients*	—	114	23.5 (±4.0)	15.2 (±3.4)	—

*Demographics*					

Gender	Male	61	19.7 (±5.1)	14.8 (±4.6)	.9902
	Female	53	28.0 (±6.2)	15.7 (±5.1)	
Age at diagnosis	≤ 19 years	62	22.1 (±5.3)	15.1 (±4.7)	.8042
	≥ 20 years	52	25.0 (±6.0)	15.4 (±5.0)	

*Disease at diagnosis*					

Primary site	Extremity	56	30.1 (±6.2)	20.4 (±5.5)	.5163*
	Pelvis	30	20.0 (±7.3)	13.3 (±6.2)	
	Central	26	15.4 (±7.1)	7.7 (±5.2)	
	Multifocal	2	0	—	
Metastases	None	61	32.4 (±6.0)	18.8 (±5.1)	< .0001
	Lung	29	24.1 (±8.0)	20.1 (±7.6)	
	Extra-pulmonary	24	0	—	

*Relapse details*					

Relapse type	Local relapse	29	37.9 (±9.0)	34.5 (±8.8)	.0189
	Distant relapse	65	20.0 (±5.0)	9.2 (±3.6)	
	Combined	20	13.3 (±8.1)	NR	
Relapse site	Local only	29	37.9 (±9.0)	34.5 (±8.8)	< .0001
	Lung ± local	40	32.1 (±7.4)	15.3 (±6.0)	
	Extra-pulmonary	45	6.7 (±3.7)	2.2 (±2.2)	
EFS1	< 18 months	75	8.0 (±3.1)	6.7 (±2.9)	< .0001
	≥ 18 months	39	53.4 (±8.1)	31.6 (±7.6)	
Worst extent of disease	Local	20	40.0 (±11.0)	35.0 (±10.7)	< .0001
	Lung	40	39.7 (±7.8)	23.3 (±6.9)	
	Extra-pulmonary	54	5.6 (±3.1)	1.9 (±1.8)	

*Relapse treatment*					

Chemotherapy or HDT	HDT	29	61.7 (±9.1)	50.6 (±9.5)	<.0001
	Chemotherapy	48	16.7 (±5.4)	4.2 (±2.9)	
	None/palliative	37	2.7 (±2.7)	NR	
Surgery and/or RT	Yes	29	51.1 (±9.4)	39.6 (±9.3)	< .0001
	No	85	14.1 (±3.8)	7.1 (±2.8)	

*2 patients with multifocal tumours are excluded from this comparison.

**Table 4:** Summary of multivariate analysis.

Variable^1^	Postrelapse survival			

	Parameter estimate	*P* (chi-sq)	Hazard ratio	95% CI

EFS1 ≥ 18 months	−1.007	< 0.001	0.365	0.22–0.59
Disease confined to local/lung at both	−1.085	< 0.001	0.338	0.22–0.52
diagnosis and relapse				
Surgery and/or radical RT to site of	−0.657	0.019	0.518	0.20–0.90
recurrence				
HDT	−1.164	< 0.001	0.312	0.17–0.58

^1^Reference: relapse within 18 months, extra-pulmonary disease at diagnosis and/or relapse, no definitive local therapy at relapse, no HDT at relapse.

**Table 5:** Outcome of patients with relapsed Ewing's tumours, derived from reports of prospective clinical trials of first-line therapy. (Abbreviations: AAW = alive and well; AWD = alive with disease; DR = distant recurrence; FU = followup; LR = local recurrence; PRS = postrelapse survival; PR = postrelapse.)

Study,	Patients at diagnosis	Median FU years (range)	Relapsed patients	Outcome after relapse	Notes
period of study,					
author					

Bacci et al [11] 1979–1995			150		—
	359	10.5	LR = 1	Overall:	
	Localised	(3.5–19)	DR = 108	8 AAW; 6 AWD	
			Both^(a)^ = 41		
ET-1 1978–1986 Craft et al [12]	142		88		—
	120 localised	11.2	LR = 26	Only 2 of 76 with	
	22 metastatic	(4.7−12)	DR = 52	metastatic relapse AAW	
			Both = 10		
CESS 86 1986–1991 Paulussen et al [13]			125	5-year PRS	– Relapse > 2 years prognosis (*P* = .0015) better 5 year PRS: 18% versus 10%
	407	6.4	LR = 22	LR: 22% (4−41%)	
	localised	(0.2−13)	DR = 92	DR: 11% (4−17%)	– Type of relapse not significant (*P* = .3095)
			Both = 11	Both: 9% (0−26%)	
NCI-86C169 1986−1992 Wexler et al [14]	54		27	All died	—
	localised 31	6.8	LR = 4		
	metastatic 23	(min3)	DR = 17		
			Both = 6		
ET2 1987−1993 Shankar et al [15]			61	11 alive^(b)^	− Relapse > 2 years better prognosis (*P* = .001)
	191	5.5	LR = 10		
	Localised	(0.2−10)	DR = 43		− Relapse site: not significant (*P* = .1)
			Both = 8		
Bacci et al [16] 1979−1996			43	42 died: 6−128 months PR 1 AAW, 12 years PR	—
	77	11	LR = 6		
	Local-pelvis	(5−23)	DR = 24		
			Both = 13		

^(a)^Defined by these authors as any patient who has ever experienced both a local recurrence and distant recurrence, regardless of the interval between them.

^(b)^Disease status is not given.

**Table 6:** Specific studies on the outcome of patients with relapsed Ewing's tumours. (Abbreviations: CHRMC = Children's Hospital and Regional Medical Center; AAW = alive and well; AWD = alive with disease; DR = distant recurrence; FLT = first-line treatment; FU = followup; LR = local recurrence; NS = not specified; Post Tr = posttreatment; PRS = postrelapse survival; LBSTS = London Bone and Soft Tissue Service; 2LT = second- line treatment.)

Study,	Patients at diagnosis	Median FU years (range)	Relapsed patients	Outcome after relapse	Factors associated with improved outcome
period of study,					
author					

St Judes 1964–1980^(a)^ Hayes et al [21]		NS	56		-NS
	78		22 in FLT	→ All died	
	localised		34 Post Tr.	→ 16 AAW, 2 AWD	
Eicess Database 1981–1990 Paulessen et al [20] (Abstract)	272	7	104	89 died	– Relapse > 2 years (10-year PRS: 0% versus 33%)
	localised			10-year PRS: 10%	
St Judes 1979–1999 Rodriguez-Galindo et al [1]	(Relapsed only)	NS	71	5-year PRS: (overall 17.7%)	– Relapse > 2 years
	41 localised		LR = 25	LR: 22% (±8%, SE)	– LR only
	30 metastasic		DR = 34	DR: 18% (±7%)	– Surgery for LR
			Both = 12	Both 13% (±8%)	– DR = lung only
					– Lung irradiation
ET-2 1985–1996 Shankar et al [2]		NS	64	Overall:	– Relapse > 2 years
	191		LR = 11	3 AAW; 4 AWD	
	localised		DR = 42	55 died^(b)^	
			Both = 11	2 lost to FU	
Rizzoli 1979–1997 Bacci et al [3]		NS	195	5-year PRS: 13.8%	
	429		LR = 1		– Relapse > 2 years
	localised		DR = 138		– Surgery or RT at relapse
			Both^(c)^ = 56		– DR = lung only
CHRMC, Seattle 1985–2002 Barker et al [22]	(Relapsed only)	NS	55	5-year PRS: (overall 20%)	
	30 localised		LR = 6	LR: 50% (95% CI, 9–91)	– Relapse > 2 years
	25 metastasic		DR = 39	DR: 20% (95% CI, 7–33)	– Response to 2LT
			Both = 10	Both: 0	– HDT at relapse
LBSTS (this report) 1992–2002	(Relapsed only)		114	5-year PRS: (overall 15.2%)	– Relapse > 18 months
	61 localised	5.6	LR = 29	LR: 35% (±9%)	– Local or lung disease only
	53 metastatic	(1.1–10.8)	DR = 65	DR: 9% (±4%)	– Surgery or RT at relapse
			Both = 20	Both: 0%	– HDT at relapse

^(a)^Includes 3 patients who had no chemotherapy during first-line treatment.

^(b)^1 patient died in a road traffic accident, apparently disease free.

^(c)^Defined by these authors as any patient who has ever experienced both a local recurrence and distant recurrence, regardless of the interval between them.
